# Skeletal muscle fiber‐type‐specific changes in markers of capillary and mitochondrial content after low‐volume interval training in overweight women

**DOI:** 10.14814/phy2.13597

**Published:** 2018-02-27

**Authors:** Rachel Tan, Joshua P. Nederveen, Jenna B. Gillen, Sophie Joanisse, Gianni Parise, Mark A. Tarnopolsky, Martin J. Gibala

**Affiliations:** ^1^ Department of Kinesiology McMaster University Hamilton ON Canada; ^2^ Pediatrics and Medicine McMaster University Hamilton ON Canada

**Keywords:** angiogenesis, capillarization, COXIV, high‐intensity interval exercise, oxidative capacity

## Abstract

High‐intensity interval training (HIIT) enhances skeletal muscle oxygen delivery and utilization but data are limited regarding fiber‐specific adaptations in humans. We examined the effect of 18 sessions of HIIT (10 × 60‐sec cycling intervals at ~90% HR_max_, interspersed by 60‐sec of recovery) over 6 weeks on markers of microvascular density and oxidative capacity in type I and II fibers in healthy but sedentary young women (Age: 26 ± 7 years; BMI: 30 ± 4 kg·m^−2^; VO_2peak_: 2.16 ± 0.45 L·m^−1^). Immunohistochemical analyses of muscle cross sections revealed a training‐induced increase in capillary contacts per fiber in type I fibers (PRE: 4.38 ± 0.37 vs. POST: 5.17 ± 0.80; main effect, *P *<* *0.05) and type II fibers (PRE: 4.24 ± 0.55 vs. POST: 4.92 ± 0.54; main effect, *P *<* *0.05). The capillary‐to‐fiber ratio also increased after training in type I fibers (PRE: 1.53 ± 1.44 vs. POST: 1.88 ± 0.38; main effect, *P *<* *0.05) and type II fibers (PRE: 1.45 ± 0.19 vs. POST: 1.76 ± 0.27; main effect, *P *<* *0.05). Muscle oxidative capacity as reflected by the protein content of cytochrome oxidase IV also increased after training in type I fibers (PRE: 3500 ± 858 vs. POST: 4442 ± 1377 arbitrary units; main effect, *P *<* *0.01) and type II fibers (PRE: 2632 ± 629 vs. POST: 3863 ± 1307 arbitrary units; main effect, *P *<* *0.01). We conclude that short‐term HIIT in previously inactive women similarly increases markers of capillary density and mitochondrial content in type I and type II fibers.

## Introduction

Interval training is characterized by intermittent periods of relatively intense exercise and recovery within a single exercise session. The method can be broadly classified into two categories: high‐intensity interval training (HIIT) involves intense but submaximal efforts that elicit ≥80% of maximal heart rate (HR_max_), whereas sprint‐interval training (SIT) refers to “supramaximal” efforts performed in an all‐out manner or at an intensity that elicits ≥100% of peak aerobic capacity (*V*O_2peak_) (Weston et al. 2014). SIT and HIIT can elicit physiological adaptations comparable to or even superior than moderate‐intensity continuous training (MICT), including indices of skeletal muscle oxygen delivery (Cocks et al. 2013) and utilization (Burgomaster et al. 2008; Little et al. 2010), despite reduced total exercise volume and time commitment.

Limited data are available regarding potential muscle fiber‐type‐specific responses to interval training in humans and most have employed SIT interventions. Scribbans et al. (2014) showed that 6 weeks of SIT, using a protocol that involved 8 × 20‐sec cycling efforts at a workload equivalent to ~170% *V*O_2peak_ with 10‐sec recovery, increased oxidative capacity in type I and type II fibers as reflected by the protein content of succinate dehydrogenase (SDH) (Scribbans et al. 2014). Cocks et al. (2016) reported similar increases in markers of capillary density in type I and II muscle fibers after 4 weeks of SIT in obese men (Cocks et al. 2016). There is some evidence of intensity‐dependent effects, as Edgett et al. (2013)showed the acute increases in markers of mitochondrial biogenesis were different when interval exercise was performed at 130% compared to 100% of maximal workload (Edgett et al. 2013).

It is possible that previously reported fiber‐specific changes in mitochondrial and capillary content following supramaximal SIT (>100% *V*O_2_peak) may not be reflective of adaptations elicited by HIIT conducted at submaximal intensities (80–100% *V*O_2_peak). Indeed, Kristensen et al. (2015) reported that an acute session of HIIT, involving 6 × 90‐sec cycling efforts at ~95% *V*O_2peak_, elicited fiber‐type‐specific responses such that glycogen utilization and AMPK expression were greater in type II versus type I fibers. This in turn could preferentially trigger divergent training adaptations, but there are no data regarding potential fiber‐type‐specific adaptations in markers of oxidative capacity or capillarization in response to a practical, low‐volume HIIT protocol. Finally, to our knowledge, no investigations into potential acute or chronic fiber‐type‐specific responses to interval training protocols have been examined in women. Given reports of a blunted and/or absent improvement in glycemic control (Metcalfe et al. 2012; Gillen et al. 2014) and mitochondrial protein synthesis (Scalzo et al. 2014) following low‐volume interval training, investigating fiber‐type‐specific responses in women may provide further information regarding sex‐based differences in the adaptive response.

The purpose of this study was to investigate fiber‐type‐specific adaptations in markers of skeletal muscle capillary and mitochondrial content in response to low‐volume HIIT in women using immunohistochemical analyses. We hypothesized that 6 weeks of low‐volume HIIT would preferentially increase markers of skeletal muscle capillarization and mitochondrial content in type II fibers.

## Materials and Methods

### Subjects

Samples were obtained from women who participated in a previously published training study (Gillen et al. 2013). To answer the novel research question posed in the present work, we analyzed muscle cross sections obtained from needle biopsies from participants in the original study (Table 1). Due to poor image quality or frozen artifact damage, we report *n *=* *10 for capillarization analyses, and *n *=* *13 for mitochondrial COXIV analyses from the original muscle dataset of *n *=* *15. Using the previously reported effect size for training‐induced adaptations in markers of capillary density (Cocks et al. 2013), our sample size was sufficient to detect a significant difference (alpha of 0.05) with 80% power (G*Power Software Inc., Kiel, Germany). Additionally, fiber‐type‐specific responses in markers of mitochondrial content following training have been investigated previously using *n *=* *10 (Scribbans et al. 2014). The study protocol was submitted to and approved by the Hamilton Health Sciences Integrated Research Ethics Board, and conformed to the guidelines outlined in the Declaration of Helsinki. Participants gave their informed written consent prior to their inclusion to the study.

**Table 1:** Subject characteristics

Characteristic	All
Fasted/Fed	7/6
Age (years)	26 ± 7
Weight (kg)	81 ± 12
Height (cm)	164 ± 7
BMI (kg·m^−2^)	30 ± 4
*V*O_2peak_ (L·min^−1^)	2.16 ± 0.45
*V*O_2peak_ (mL·kg^−1^·min^−1^)	27.4 ± 6.5

### Exercise training

Participants trained three times per week for 6 weeks as previously described (Gillen et al. 2013). Briefly, each session involved 10 × 60‐sec cycling efforts at a workload average of ~85% W_max_, that elicited ~90% of HR_max_ interspersed by 60‐sec of active recovery at 50 W. The training stimulus was relative to each participants HR_max_, as determined by an incremental exercise test to exhaustion (Gillen et al. 2013). Heart rate was monitored continuously at each training session and exercise workload was increased as needed (in 5 or 10 W increments) to maintain the desired relative exercise intensity. Owing to a lack of difference between fasted and fed groups in exercise training intensity and physiological adaptations (Gillen et al. 2013), groups were combined for the purpose of the present investigation.

### Muscle biopsy

Resting needle muscle biopsies were obtained before (PRE) and 96 hr following (POST) the last training bout, as previously described (Gillen et al. 2013). One piece of muscle was immediately snap frozen in liquid nitrogen. A second piece of each muscle sample was mounted in Optimum Cutting Temperature compound (Tissue‐Tek; Sakura Finetek, Torrance, CA) and frozen in isopentane cooled with liquid nitrogen. All samples were subsequently stored at −80°C.

### Immunohistochemical analysis

Muscle cross sections (7 *μ*m) were prepared from embedded sections and immunohistochemical analyses for markers of capillarization were conducted using methods previously described (Nederveen et al. 2016). For determination of fiber‐type‐specific capillarization, muscle cross sections were stained against antibodies CD31 (1:30; ab28364; Abcam, Cambridge, MA, CA); biotinylated goat anti‐rabbit IgG (1:200; Vector Laboratories, Inc., Burlingame, CA); MHCII (MHCII; fast‐isoform; ab91506; Abcam) diluted in MHCI (MHCI; slow‐isoform, neat; 1:1000; DSHB); and laminin (1:500, ab11575; Abcam). Samples were incubated for 2‐h at room temperature using secondary antibodies streptavidin‐594 fluorochrome (1:100; Invitrogen, Molecular Probes, Carlsbad, CA); MHCII (Alexa Fluor 647‐R, 1:500) and MHCI (Alexa Fluor 488‐M, 1:500; Invitrogen, Molecular Probes). Nuclei were visualized with 4′, 6‐diamidino‐2‐phenylindole (DAPI, 1:20,000; Sigma‐Aldrich, Oakville, ON, Canada). For determination of mitochondrial content, fiber‐type‐specific COXIV protein intensity was measured. Slides were stained with antibodies against COX subunit IV (COXIV; 1:400; MS408, Mitosciences, Eugene, OR); laminin (1:500, ab11575; Abcam) and myosin heavy chain type II (MHCII; fast‐isoform; 1:1000, ab91506; Abcam). Samples were incubated for 2‐h at room temperature using secondary antibodies for laminin (Alex Fluor 647, 1:500); COXIV (Alexa Fluor 488) and MHCII (Alexa Fluor 647‐R, 1:500). Indices of skeletal muscle capillarization were performed as described by Hepple et al. (1997), which included capillary‐to‐fiber ratio on an individual fiber basis (C/F_*i*_), capillary contacts, and capillary density. Analyses were performed blinded on 50 fibers/subject/time point/fiber‐type. COXIV protein intensity was quantified for 25 fibers/subject/time point/fiber‐type, and an average was determined. Absolute values are reported, and also normalized to PRE for each subject to be shown as a fold change.

Images were acquired from a Nikon Eclipse T*i* microscope at 20× magnification and captured with a Photometrics CoolSNAP HQ2 fluorescent camera (Nikon Instruments, Melville, NY). Images were captured and analyzed manually using the Nikon NIS Elements AR 3.0 software, with the same image capture settings for each subject (Nikon Instruments).

### Western blotting

A second piece of muscle was homogenized as described previously (Little et al. 2010; Gillen et al. 2013). Western blot analyses were performed for COXIV (COX subunit IV MS408, Mitosciences) based on previously optimized conditions described elsewhere (Little et al. 2011).

### Statistical analysis

A two‐way repeated measures analysis of variance (time × fiber‐type) was used to examine changes in C/F_*i*_, capillary contacts, COXIV protein intensity, and fiber area. Whole‐muscle capillary density and COXIV protein content were analyzed using a paired Student's t‐test (pre vs. posttraining). All analyses were performed using SPSS Statistics Software (Version 22.0; IBM Corp, Armonk, NY) and the level of significance was set at *P* ≤ 0.05. All data are presented as means±SD.

## Results

### Exercise training descriptives

All subjects completed the 18 supervised exercise sessions over 6 weeks. Heart rate elicited at the end of each interval was on average 92 ± 1% of HR_max_. Average interval intensity was 166 ± 55 W, which corresponded 86 ± 6% of subject's baseline maximal workload. Following training, there was an 18% increase in *V*O_2peak_ (27 ± 6.5 vs. 32 ± 5.6, *P *<* *0.05), which corresponded with a 13% increase in peak power output (207 ± 34 vs. 234 ± 33, *P *<* *0.05).

### Fiber area

There was no change in fiber area following training in type I fibers (PRE: 6052 ± 1155 vs. POST: 7476 ± 2522, *P *>* *0.05) or type II fibers (PRE: 6637 ± 1479 vs. POST: 7181 ± 2360, *P *>* *0.05).

### Capillarization

**Figure 1:**
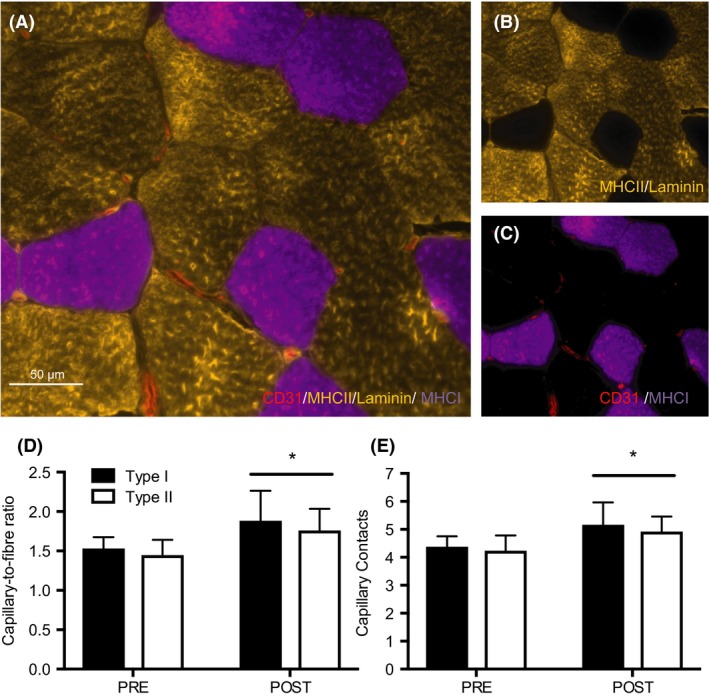
Capillary expression in type I and II muscle fibers after 6 weeks of HIIT. Representative image of a CD31/MHCII/laminin/MHCI stain (A). Co‐expression of MHCII/laminin (gold) (B), and CD31/MHCI (red/purple) (C). Capillary‐to‐fiber ratio (D) and capillary contacts (E) before (PRE) and after (POST) training in type I and type II fibers. Data are presented as means ± SD. *P <* *0.05, main effect of time. HIIT, high‐intensity interval training.

Representative images are presented in Figure 1A–C. The C/F_*i*_ ratio increased similarly after HIIT in type I (PRE: 1.53 ± 1.44 vs. POST: 1.88 ± 0.38) and type II fibers (PRE: 1.45 ± 0.19 vs. POST: 1.76 ± 0.27, main effect for time, *P *<* *0.05, no interaction effect, *P *>* *0.05, Fig. 1D). Capillary contacts increased in both type I (PRE: 4.38 ± 0.37 vs. POST: 5.17 ± 0.80) and type II fibers (PRE: 4.24 ± 0.55 vs. POST: 4.92 ± 0.54, main effect of time, *P *<* *0.05, no interaction effect, *P *>* *0.05, Fig. 1E) following training. There was no difference in CC or C/Fi between fiber types at either time point (*P *>* *0.05). Changes in whole‐muscle capillary density were not detected in response to HIIT (150 ± 47 vs. 177 ± 55 cap·mm^−2^, *P *>* *0.05, Fig. 3A).

### Mitochondrial content

**Figure 2:**
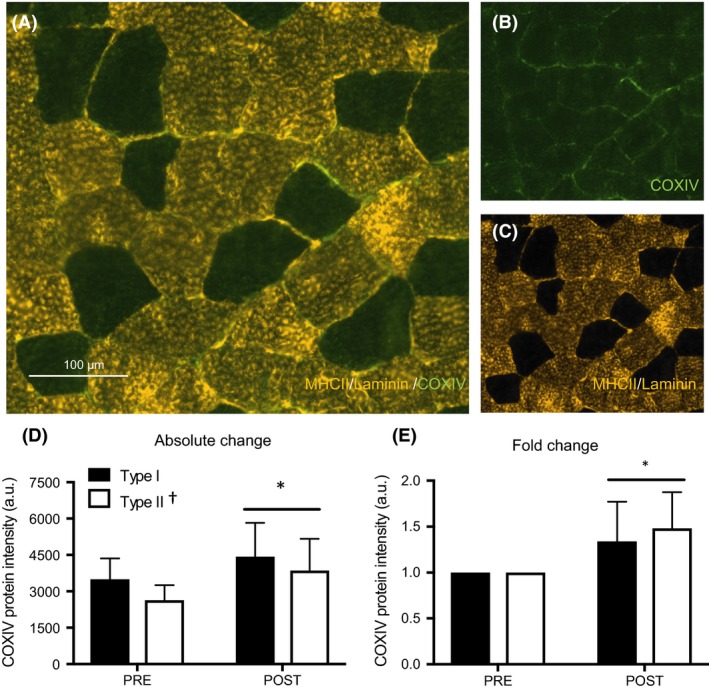
COXIV expression in type I and II muscle fibers after 6 weeks of HIIT. Representative image of a MHCII/laminin/COXIV stain (A). Single channel views of COXIV (green) (B), and co‐expression of MHCII/laminin (gold) (C). Absolute (D) and relative (E) COXIV expression before (PRE) and after (POST) training in type I and type II fibers. Data are presented as means ± SD. *P <* *0.05, main effect of time; †*P *<* *0.05, main effect of fiber type. HIIT, high‐intensity interval training.

**Figure 3:**
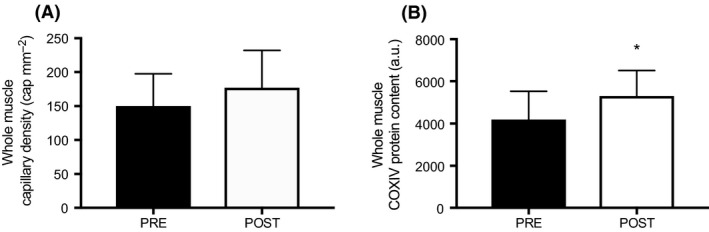
Whole‐muscle capillary density (A) and COXIV protein content (B) before (PRE) and after (POST) 6 weeks of HIIT. *P <* *0.05, pre versus post. HIIT, high‐intensity interval training

Representative images are presented in Figure 2A–C. Fiber‐type‐specific analyses revealed higher COXIV protein intensity in type I compared to type II fibers (3972 ± 1223 vs. 3247 ± 1186 arbitrary units (AU); *P *<* *0.0001, Fig. 2D). Following training, COXIV protein intensity increased by 27% in type I fibers (PRE: 3500 ± 858 vs. POST: 4442 ± 1377 AU, Fig. 2D) and by 46% in type II fibers (PRE: 2632 ± 629 vs. POST: 3863 ± 1307 AU, main effect of time, *P *<* *0.01, no interaction effect, *P *>* *0.05, Fig. 2D). When absolute COXIV protein intensity was normalized to baseline and presented as a fold change, we observed a 1.34‐ and 1.48‐fold increase in type I and type II fibers, respectively (main effect of time, *P *<* *0.01, time x fiber‐type interaction, *P *=* *0.086, Fig. 2E). At the whole‐muscle level, there was a ~27% increase in COXIV protein content following HIIT (PRE: 4188 ± 1341 vs. POST: 5304 ± 1213 AU; *P *<* *0.05, Fig. 3B).

## Discussion

The major novel finding from this study was that 6 weeks of low‐volume HIIT in previously inactive women increased markers of oxygen delivery and utilization similarly in type I and II muscle fibers. Specifically, we assessed markers of skeletal muscle oxidative capacity including capillary contacts, C/F_*i*_, capillary density, as well as COXIV protein content to reflect mitochondrial content. To our knowledge, this is the first study to examine fiber‐type‐specific responses to low‐volume HIIT in a cohort of women. Contrary to our hypothesis, our findings reveal that low‐volume HIIT induces similar skeletal muscle microvascular and mitochondrial adaptations in type I and type II muscle fibers, at least when measured after 6 weeks of training.

It is well documented that low‐volume interval training increases skeletal muscle oxidative potential at the whole‐muscle level (MacInnis and Gibala 2016). Central to this improved oxidative capacity is greater microvascular and mitochondrial density, which is imperative for the delivery and utilization of oxygen to support aerobic metabolism (Bassett and Howley 2000). While many studies have revealed such adaptations at the whole‐muscle level following low‐volume interval training in both men and women (Little et al. 2011; Cocks et al. 2013; Gillen et al. 2013), limited data are available on fiber‐type‐specific adaptations. Given the greater activation of type II fibers with increasing exercise intensity (Krustrup et al. 2004; Edgett et al. 2013), it has been hypothesized that oxidative remodeling may preferentially occur in type II fibers. Current investigations are limited, however, and importantly, have only been conducted in men (Cocks et al. 2013) or mixed cohorts of men and women (Scribbans et al. 2014). Thus, we sought to investigate skeletal muscle oxidative remodeling at the fiber‐specific level following low‐volume HIIT in women.

With respect to the capacity for oxygen delivery, we found similar increases in markers of capillarization in type I and type II fibers following 6 weeks of low‐volume HIIT. In response to training, capillary contacts and C/F_*i*_ increased by 17 and 22%, respectively, with no difference between fiber types. These findings are consistent with recent investigations in men, which have reported similar increases in indices of capillary growth among fiber types following 4–6 weeks of SIT (Cocks et al. 2016). We also observed an 18% improvement in whole‐muscle capillary density, albeit not statistically significant. The relative increase (18%) is consistent with previous low‐volume SIT interventions in men (Cocks et al. 2013, 2016; Scribbans et al. 2014), however, our cohort of women displayed large variability in the adaptive response, potentially limiting our ability to detect significance.

Microvascular remodeling is stimulated by shear stress, hypoxic environments, and metabolic demand (Egginton 2009), all of which are exacerbated in type II fibers during high‐intensity exercise (Krustrup et al. 2004). However, consistent with previous reports in men (Cocks et al. 2016), adaptations in markers of capillary density were similar among fiber types following 6 weeks of low‐volume HIIT in women. While a direct comparison to MICT was not made in this study, previous investigations have reported similar remodeling of type I and II fibers following short‐term SIT and MICT (Scribbans et al. 2014; Cocks et al. 2016) reinforcing that there does not appear to be preferential vascular remodeling of type II fibers following low‐volume interval training. Vascular endothelial growth factor (VEGF) is a proangiogenic factor considered to be central to capillary growth in response to exercise training (Egginton 2009). VEGF gene expression is increased similarly in men and women following an acute bout of low‐volume SIT (Skelly et al. 2017), which may underpin the comparable increase in capillary density between sexes in our study and those previous (Cocks et al. 2013). It has been suggested that the duration of shear stress exposure may outweigh the magnitude of shear stress for enhancing capillary networks (Egginton 2009), and thus low‐volume HIIT may provide an insufficient stimulus for more robust vascular remodeling in type II fibers. Alternatively, it could also be postulated that a longer HIIT intervention is required to observe fiber‐type‐specific differences through the sprouting of new microvascular vessels in type II fibers, which seems to require longer periods of training (Hoier and Hellsten 2014).

With respect to mitochondrial capacity, we found similar increases in the protein content of COXIV in type I and II fibers following training. Exercise intensity is a critical determinant of skeletal muscle mitochondrial remodeling (Bishop et al. 2014; MacInnis and Gibala 2016) and we have previously reported improved mitochondrial capacity at the whole‐muscle level following low‐volume HIIT in women (Gillen et al. 2013). It has been suggested that the greater motor unit recruitment during high‐intensity exercise (Sale 1987), and subsequent remodeling of type II fibers, could largely mediate the positive relationship between exercise intensity and mitochondrial adaptations. However, few studies have investigated fiber‐specific mitochondrial responses to low‐volume HIIT, with equivocal results reported in the literature. For example, four decades ago, 8 weeks of HIIT was shown to induce greater absolute increases in SDH activity in type II compared to type I fibers (Henriksson and Reitman 1976). More recently, an acute bout of interval exercise involving 6 × 90‐sec sprints at ~95% *V*O_2peak_ with 150‐sec recovery induced greater glycogen utilization and AMPK expression in type II versus type I fibers (Kristensen et al. 2015). This in turn could preferentially trigger mitochondrial adaptations in type II fibers over the course of training. However, recent investigations in men do not support this hypothesis as similar increases in SDH (Scribbans et al. 2014) and COXIV (Shepherd et al. 2013; Christensen et al. 2015) expression have been reported after SIT in type I and II fibers. Our data extend these findings to women, revealing similar mitochondrial remodeling type I and type II fibers following low‐volume HIIT. However, given the rapid response to low‐volume HIIT (Little et al. 2010; Vincent et al. 2015) it is possible that there is a preferential remodeling of type II fibers early in a training program, which may not be apparent by 6 weeks. Future research investigating the time course of mitochondrial remodeling in type I and type II fibers following low‐volume HIIT is needed to provide insight to fiber‐type‐specific adaptations to training.

We acknowledge limitations associated with this study, including the lack of differentiation between type IIa and type IIx fiber pools. However, previous investigations have reported no differences in the adaptive response among type IIa and IIx fibers in response to low‐volume interval training in men (Scribbans et al. 2014). In addition, although our methodology is similar to those employed by others (Cocks et al. 2013; Scribbans et al. 2014), immunofluorescent staining may not be sensitive enough to detect the relatively small training‐induced differences between fiber types. Furthermore, menstrual cycle phase was not controlled for in this study, and oral contraceptive (OC) use was reported for some (*n *=* *3) but not all subjects. While we were not powered to detect differences as a result of OC‐use, SIT‐induced improvements in cardiorespiratory fitness are reportedly blunted in OC‐users (Schaumberg et al. 2017). Future research is needed to clarify the effect of OC‐use on skeletal muscle adaptations to low‐volume interval training. Lastly, we examined fiber‐type‐specific adaptations in women alone. While this is a critical step for improving the disparity of exercise‐induced muscle remodeling research in women (Costello et al. 2014), a direct sex comparison would yield additional insight and warrants future attention.

In summary, we provide the first report of fiber‐type‐specific responses to low‐volume HIIT in women. Consistent with previous findings in men (Shepherd et al. 2013; Scribbans et al. 2014; Cocks et al. 2016), we observed similar increases in markers of capillary and mitochondrial content in type I and type II fibers following 6 weeks of training. Our findings suggest that both type I and type II fibers contribute to the improvements in oxidative capacity at the whole‐muscle level, at least when measured following 6 weeks of training. Future research is needed, using methodological advances for fiber‐type‐specific analyses (Murphy 2011) to investigate the time course of mitochondrial and vascular remodeling in type I and type II fibers following low‐volume interval training in both men and women.

## Conflict of Interest

The authors have no conflicts of interest to declare.
